# Differential Oral Microbial Input Determines Two Microbiota Pneumo‐Types Associated with Health Status

**DOI:** 10.1002/advs.202203115

**Published:** 2022-08-28

**Authors:** Jingxiang Zhang, Yiping Wu, Jing Liu, Yongqiang Yang, Hui Li, Xiaorong Wu, Xiaobin Zheng, Yingjian Liang, Changli Tu, Meizhu Chen, Cuiyan Tan, Bozhen Chang, Yiying Huang, Zhengguo Wang, Guo‐Bao Tian, Tao Ding

**Affiliations:** ^1^ Department of Immunology and Microbiology Zhongshan School of Medicine Sun Yat‐sen University Guangzhou 510080 China; ^2^ Key Laboratory of Tropical Diseases Control (Sun Yat‐sen University) Ministry of Education Guangzhou 510080 China; ^3^ Department of Respiratory Medicine The Fifth Affiliated Hospital of Sun Yat‐sen University Zhuhai 519000 China; ^4^ School of Medicine Xizang Minzu University Xianyang Shaanxi 712082 China

**Keywords:** lung microbiota, respiratory microbiome, lung function, oral microbiota, neutral model, cytokines

## Abstract

The oral and upper respiratory tracts are closely linked anatomically and physiologically with the lower respiratory tract and lungs, and the influence of oral and upper respiratory microbes on the lung microbiota is increasingly being recognized. However, the ecological process and individual heterogeneity of the oral and upper respiratory tract microbes shaping the lung microbiota remain unclear owing to the lack of controlled analyses with sufficient sample sizes. Here, the microbiomes of saliva, nasal cavity, oropharyngeal area, and bronchoalveolar lavage samples are profiled and the shaping process of multisource microbes on the lung microbiota is measured. It is found that oral and nasal microbial inputs jointly shape the lung microbiota by occupying different ecological niches. It is also observed that the spread of oral microbes to the lungs is heterogeneous, with more oral microbes entering the lungs being associated with decreased lung function and increased lung proinflammatory cytokines. These results depict the external shaping process of lung microbiota and indicate the great value of oral samples, such as saliva, in monitoring and assessing lung microbiota status in clinical settings.

## Graphical abstract

This study provides a quantitative description of the shaping process of the lung microbiota. Oral and nasal microbes jointly shape the lung microbiota by occupying different ecological niches. There are significant interindividual variations in the spread of oral microbes to the lungs, with more of oral microbes entering the lungs associated with decreased lung function and increased pro‐inflammatory cytokines in the lungs.

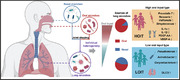

## 1 Introduction

The lungs were once believed to be sterile. In recent years, however, metagenomic studies have confirmed the presence of commensal bacteria in the lungs of healthy individuals.^[^ ^1^, ^2^, ^3^ ^]^ This may be implicated in training host immunity, particularly the respiratory system,^[^ ^1^, ^4^, ^5^ ^]^ but their origins are controversial.^[^ ^6^, ^7^ ^]^ The lungs and lower respiratory tract are linked to the upper respiratory tract and oral cavity, and several studies have reported that microbes in the nasal and oral cavities are involved in the formation of the lung microbiota. However, this process remains unclear due of the lack of quantitative data and explanations for individual variations.^[^ ^8^, ^9^, ^10^ ^]^

Interactions between the microbiota across different body sites have been reported,^[^ ^11^, ^12^, ^13^ ^]^ with oral and intestinal microbiota interactions gaining considerable traction in recent years.^[^ ^14^, ^15^, ^16^, ^17^, ^18^ ^]^ Disruption of the oral microbiome has been reported to be a risk factor for enteritis, arthritis, and lupus erythematosus.^[^ ^14^, ^15^, ^16^ ^]^ Interestingly, accumulated oral taxa, such as *Fusobacterium nucleatum*, have been identified in the gut of patients with colorectal cancer.^[^ ^17^, ^18^ ^]^ Ding et al. reported that oral and intestinal microbiota were not comparable in composition, but microbial community types were mutually predictable between the sites.^[^ ^19^ ^]^ Based on these studies, researchers have recently identified clinically significant interactions between oral and lung microbiota. Dickson et al. found that microbes from the *Prevotella*, *Streptococcus*, *Veillonella*, and *Neisseria* genera, which were previously common in oral spaces, were also dominant in the lungs of healthy individuals.^[^ ^8^, ^20^, ^21^ ^]^ In addition, Segal et al. observed that lung microbiota enrichment with oral taxa was related to Th17 cell proliferation, the upregulation of inflammatory pathways in the lungs, and poor prognoses in patients with lung cancer.^[^ ^9^, ^22^, ^23^ ^]^ The upper and lower airways are anatomically close together, and bacteria can travel to the lungs through the air and nasal secretions.^[^ ^24^ ^]^ In recent years, accumulating evidence has indicated the role of bacterial communities in the upper respiratory tract in preventing respiratory pathogens from infecting mucosal surfaces and spreading to the lower respiratory tract. For most respiratory bacterial pathogens, the upper respiratory tract must be colonized before causing upper respiratory tract, lower respiratory tract, or disseminated respiratory tract infections.^[^ ^25^, ^26^ ^]^

The main sampling methods used to study lung microbiota include sputum samples,^[^ ^27^ ^]^ bronchial aspirates,^[^ ^28^ ^]^ bronchoalveolar lavage fluid (BALF).^[^ ^20^ ^]^ and endobronchial biopsies.^[^ ^29^ ^]^ While these methods infer or directly measure lung microbiota composition, they are easily contaminated and contain low biomass.^[^ ^30^ ^]^ Therefore, the characterization of lung microbiome communities remains challenging. Although lung microbiota research has advanced considerably in recent years, it remains largely limited by technical difficulties, including the collection of invasive BAL specimens from patients and the isolation, culturing, and sequencing of microbes from low‐biomass samples. Given the connection and biomass exchange between the oral cavity and respiratory tract, oral specimens such as saliva may provide highly optimal solutions for lung microbiota analysis. However, to assess the rationality of this approach, the extent to which oral and upper respiratory tract microbiota variability contributes to lung microbiota heterogeneity must be evaluated.

To answer this question and to systematically study the role of upstream sources such as oral and nasal cavities on the formation of lung microbiota, we enrolled 67 patients with lung cancer and 32 volunteers with nonmalignant lung diseases and collected their saliva, nasal swabs, oropharyngeal swabs, and BAL samples. We integrated metagenomic sequencing and cytokine profiling with detailed clinical information to analyze the systemic association between oral and lung microbiota. We found that oral microbes contributed to most of the dominant taxa in the lungs; however, these contributions were highly heterogeneous. This individual heterogeneity shaped the unique oral and lung microbiomes, the characteristics of which are closely associated with host lung health.

## 2 Results

### 2.1 Oral and Nasal Microbes Contribute to the Lung Microbiome by Occupying Different Ecological Niches

**Figure 1:**
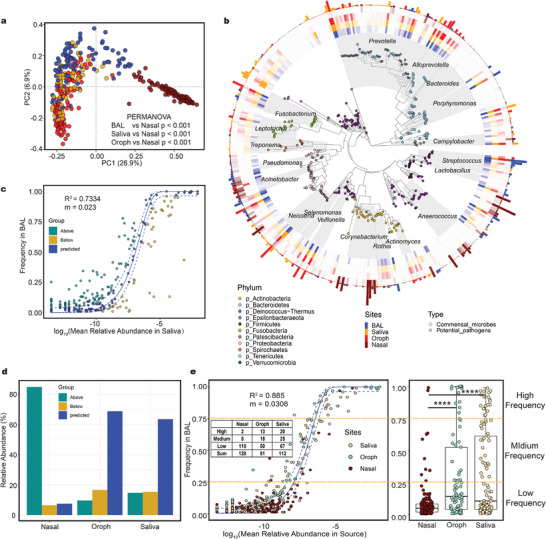
The taxonomic composition of various type samples and the results of neutral model fitting. a) PCoA based on Bray‐Curtis distance demonstrated that the community structures of the saliva (*n* = 81), oropharynx (*n* = 87) and BAL (*n* = 99) were similar, but the community composition of the nasal (*n* = 86) samples was specific. b) The phylogenetic tree of ASVs of which the shape of the tip points indicate the types of microbes (commensal microbes or potential pathogens). The transparency of the heatmap indicates the abundance of microbes, and the colors of the heatmap indicate different sites of the human body. The bar plot indicates the relative abundance of the most prevalent species at the body sites. c) Results of neutral model fitting with saliva as source. The solid blue line represents the fitting curve and the dashed blue line represents the 95% confidence interval. The coefficient of determination (*R* ^2^) was the goodness of fit of the neutral model. It ranged from ≤0 (no fit) to 1 (perfect fit). d) The relative abundance of bacteria in the neutral models. e) Results of multiple sources neutral model fitting. The colors of the dots represents the body sites that provided the bacteria. Bacteria were grouped according to their frequency in lungs (upper and lower quartiles). The numbers in the table represents the numbers of taxa contributed by each body site in each group. PCoA: principal coordinate analysis; PERMANOVA: permutational multivariate analysis of variation. Box plot: centerline, median; box limits, upper and lower quartiles; error bars, 95% CI. Differences between groups were assessed using Wilcoxon test, * *p* < 0.05, ** *p* < 0.01, *** *p* < 0.001, **** *p* < 0.0001.

We profiled the microbial compositions of the BAL, nasal, saliva, and oropharyngeal swab samples from 16s rRNA gene sequencing data and calculated the similarities among these samples as the Bray‐Curtis distance. These similarities were visualized on a principal coordinate plot (PCoA), which showed that the microbiota compositions of the BAL, saliva, and oropharynx were clearly separated from that of the nasal cavity in the first axis (**Figure** **1**a, PERMANOVA, *p <* 0.001). The most abundant genera in the BAL samples included *Streptococcus* (mean ± SD, 37.5% ± 15.7%), *Neisseria* (14.41% ± 6.93), *Prevotella 7* (13.85% ± 5.16), and *Haemophilus* (12.3% ± 10.8) (Figure 1b), consistent with previous research.^[^ ^2^, ^8^, ^9^ ^]^ We used the 16sPIP^[^ ^31^ ^]^ database to identify potentially pathogenic amplicon sequence variants (ASVs) and detected large numbers from the *Prevotella 7*, *Streptococcus*, and *Neisseria* genera (Figure 1b). The microbiota composition of the nasal swabs was distinct from that of the other three sample types; the dominant genera included *Corynebacterium* (33.5% ± 20.15), *Staphylococcus* (25.1% ± 22.14), and *Anaerococcus* (3.84% ± 5.36).

Next, we used a neutral model to gauge the relative contribution of the upper respiratory tract and oral microbiome to the lung microbiome assembly (details of the neutral model are provided in the Supporting Information). The goodness‐of‐fit (*R* ^2^) values of the neutral model were 0.7334 for saliva, 0.714 for the oropharynx, and −0.0987 for the nasal cavity (*R* ^2^ ≤ 0, no fit; *R* ^2^ = 1, perfect fit) (Figure 1c (saliva), Figure S2a (nasal and oropharyngeal), Supporting Information). We observed that the most common bacteria in the BAL (including *Prevotella 7*, *Neisseria*, *Streptococcus*, *Veillonella*, *Haemophilus*, *Fusobacterium*, and *Allprevotella*) fitted into the saliva‐BAL neutral model, suggesting that the dominant bacteria in the lungs were mostly provided by the oral cavity (95% Wilson score interval, Figure 1d and Figure S2b, Supporting Information). Based on neutral model theory, these results suggest that oral and oropharyngeal microbes are the main shaping sources of lung microbiota, while the entry of nasal microbes into the lung may have been impeded by host and environmental factors (Figure 1d and Figure S2b, Supporting Information).

Next, we combined saliva, oropharyngeal, and nasal swab microbiota as a refitted neutral model input and constructed a multiple source neutral model to assess the contribution of bacteria from various sources to lung microbiota assembly (Figure 1e). Similar to the results of the single‐source model, the oral cavity and oropharynx still contributed most of the high‐frequency bacteria to the multiple‐source neutral model. Although most of the nasal contributing species were detected less frequently in the lungs, the number of contributing species was highest here (128) (Figure 1e). The results of this multiple‐source neutral model suggest that the formation of the lung microbiota is a multisource process. The oral cavity and oropharynx contributed to the major component of the lung microbiota, whereas the nasal cavity provided less common and less abundant organisms.

### 2.2 Oral Bacterial Input into the Lungs Shapes Two Lung Microbiota Community Types

**Figure 2:**
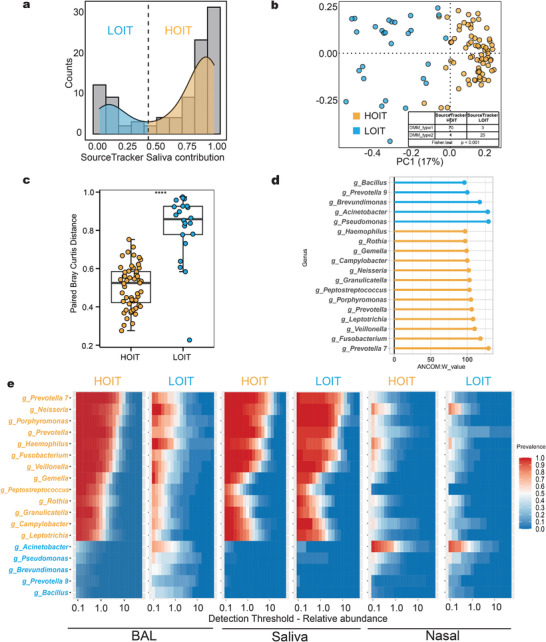
The definition and microbiome characteristics of the pneumo‐types. a) SourceTracker was used to calculate the contribution value of saliva to BAL and show the kernel density map and frequency distribution histogram of the contribution value. The nuclear density map showed a bimodal distribution. b) PCoA based on Bray‐Curtis demonstrated that BAL samples of HOIT (*n* = 71) clustered separately from LOIT (*n* = 28) BAL samples. The contents of the table showed the distribution of results for the two methods. The four samples with inconsistent classification results are indicated by red circles in the figure. c) The paired Bray‐Curtis distances between the BAL and saliva samples between the pneumo‐types. d) ANCOM and LEfSe demonstrated distinct genera between the pneumo‐types. The larger the W value in ANCOM results, the more significant the difference between the pneumo‐types. e) Core microbiota heatmaps showing abundance of taxa and prevalence across difference samples from the LOIT and HOIT. Taxa listed were selected on the basis on the LEfSe and ANCOM results. ANCOM: analysis of composition of microbiomes; LEfSe: Linear discriminant analysis Effect Size. Box plot: centerline, median; box limits, upper and lower quartiles; error bars, 95% CI. Differences between pneumo‐types were assessed using Wilcoxon test, * *p* < 0.05, ** *p* < 0.01, *** *p* < 0.001, **** *p* < 0.0001.

Saliva samples were selected as representatives of the oral cavity and oropharynx in subsequent analyses because the oral cavity and oropharynx play a similar role in shaping the lung microbiota. To quantitatively measure the communication intensity of oral and lung microbiota in individuals, we used SourceTracker^[^ ^32^ ^]^ to calculate saliva microbial contributions to the lung microbiota. Interestingly, the scores of oral microbes contributing to the lung microbiota followed a bimodal distribution (**Figure** **2**a), suggesting that communications could be categorized as high‐ and low‐intensity modes. To further investigate how these modes impacted the lung microbiota, we used the trough value of this contribution score as the cut‐off to define the two microbiota types and compare their differences (Figure 2a). The two pneumo‐types were defined as the high‐oral‐input type (HOIT) and low‐oral‐input type (LOIT) based on the contribution of oral bacteria to the lung microbiota. To verify the rationality and robustness of this categorization, we also applied the Dirichlet multinomial mixture (DMM)^[^ ^33^ ^]^ model approach to the BAL data and identified two lung microbiota community types (Figure S3a, Supporting Information). Although the algorithms and required input data were different, our results were highly consistent between the two methods (Figure 2b and Table S1, Fisher's test *p* < 0.001, Supporting Information). Finally, we compared the distance between paired saliva and BAL samples and found that the distance in the HOIT was significantly lower than that in the LOIT (Figure 2c, Bray‐Curtis distance, Wilcoxon test *p* < 0.0001).

To verify that pneumo‐type was the most important explanatory factor for lung microbiome heterogeneity in our cohort, we assessed all factors for lung microbiome differences using variance partitioning analysis (VPA). We found that the pneumo‐type had a higher explanatory power for the lung microbiome than the other factors (Figure S3b, VPA: *R^2^* = 10.2, Supporting Information). We observed a significant difference in composition between the microbiotas of the two pneumo‐types (Figure S3d, Bray‐Curtis distance, PERMANOVA, *p <* 0.001, Supporting Information). Alpha diversity was significantly higher in the HOIT group (Figure S3c, Shannon index, Wilcoxon test *p* = 0.0034, Supporting Information). We also compared the lung microbiome differences between patients with lung cancer and healthy volunteers. We observed no significant differences in microbiota alpha diversity (Figure S3e, Wilcoxon test *p* > 0.05, Supporting Information) or microbiota composition between patients with lung cancer and volunteers (Figure S3f, PERMANOVA *p* > 0.05, Supporting Information). In addition, we found no significant differences in pneumo‐type distribution between lung cancer patients and volunteers (chi‐square test, *p* > 0.05). Overall, the pneumo‐type was the most important explanatory factor for the lung microbiome in our study. Thus, we focused on the differences between the two pneumo types in subsequent analyses.

To further demonstrate that pneumo‐type universality is prevalent across different populations, we chose two public datasets for validation (PRJNA269493 and PRJNA316098). We constructed a random forest model classifier based on our own data to classify the other two datasets (Figure S4a,b, Supporting Information). The results of the external datasets showed a pneumo‐type composition similar to that of our data (Figure S4c–f, Supporting Information).

Eighteen genera were differentially enriched in either the HOIT or LOIT groups (Table S2, Supporting Information). *Prevotella 7*, *Fusobacterium*, and *Veillonella* genera were significantly enriched in the HOIT, and *Pseudomonas*, *Acinetobacter*, and *Brevumdionas* were significantly enriched in the LOIT (Figure 2d). For the 55 BAL samples that contained sufficient biomass materials for downstream analyses, shotgun metagenomic sequencing indicated that *Prevotella melaninogenica, Neisseria subflava*, and *Neisseria mucosa* were significantly enriched in the HOIT (Table S2, Supporting Information). Some genera enriched in the HOIT were commonly considered oral space inhabitants (e.g., *Prevotella 7*, *Neisseria*, and *Veillonella*), while genera enriched in the LOIT (e.g., *Pseudomonas*, *Acinetobacter*, and *Corynebacterium 1*) were rarely seen in the oral space but had a high prevalence rate and abundance in the nasal cavity (Figure 2e).

### 2.3 Community Assembly Characteristics and Microbial Function Differences of Pneumo‐types

**Figure 3:**
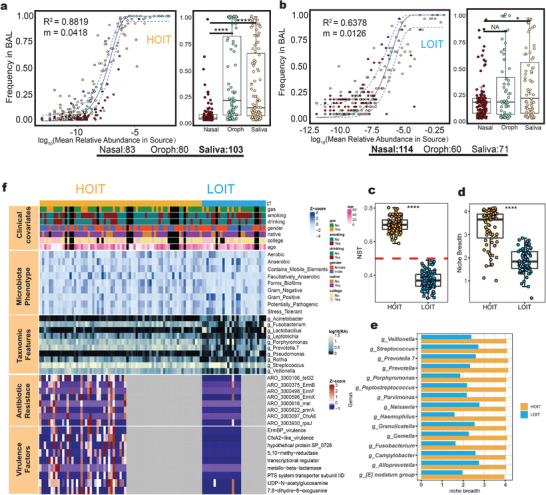
Lung community assembly and microbial functional characteristics. a,b) Results of multiple sources neutral model fitting of HOIT (*n* = 71) or LOIT (*n* = 28). c) NTS were randomized 100 times to compare the proportion of random processes in the lung community assembly process. d) The top 15 taxa with the highest niche breadth in the HOIT showed significant differences in niche breadth between the pneumo‐types. f) Top‐to‐bottom modules of the heatmap represent clinical phenotype annotations, microbiota phenotypes, genus characteristics, resistant genes, and virulence genes, respectively. Box plot: centerline, median; box limits, upper and lower quartiles; error bars, 95% CI; NST: normalized stochasticity ratio. Differences between pneumo‐types were assessed using Wilcoxon test, * *p* < 0.05, ** *p* < 0.01, *** *p* < 0.001, **** *p* < 0.0001.

A neutral model was applied to evaluate the community assembly process. In both pneumo‐types, the oral and lung microbiota communication process fit the neutral model (both *R* ^2^ values > 0), while the coefficient of determination (*R* ^2^) and diffusion coefficient (m) for the LOIT (*R* ^2^ = 0.6379, m = 0.0126) (**Figure** **3**a) was less than that of the HOIT (*R* ^2^_HOIT_ = 0.8819, m = 0.0418) (Figure 3b), which indicated that the LOIT population had more constraints on the spread of microbes from the oral and upper respiratory tract to the lungs. The number of species contributing to the nasal cavity in the LOIT group was significantly higher than that in the HOIT group, suggesting that nasal cavity microbes also play key roles in shaping the lung microbiota, especially in the LOIT population. We further used the normalized stochasticity ratio (NST), which assesses the proportion of random processes in community assembly, and observed a higher proportion of random processes in the HOIT (Figure 3c). The niche breadth of bacteria,which represents the prevalence and relative abundance of bacteria in the BAL samples, was significantly higher in the HOIT than in the LOIT (Figure 3d). The niche breadth of dominant oral bacteria, such as *Veillonella*, *Prevotella 7* and *Streptococcus* in the lungs was significantly different between the pneumo‐types (Figure 3e). These results collectively reflect the differences in community assembly between the pneumo‐types.

We further explored the lung microbial phenotypes and functions of each pneumo‐type. We used Bugbase^[^ ^34^ ^]^ to predict organism levels in the microbiome phenotypes of the lung microbiome (Figure 3f). A higher proportion of facultative anaerobes in the lung microbiome and a stronger potential to synthesize biofilms in the HOIT were observed when compared with the LOIT (Figure S5a, Supporting Information). Using the ShortBRED method, we also observed that a large number of antibiotic resistance and virulence genes were enriched in the HOIT; most antibiotic‐resistant genes were macrolide‐resistant (*ErmB*, *ErmF*, *ErmX*, and *mel*) (Figure 3f). These findings are consistent with those of a previous study that showed that the core respiratory resistome was composed of macrolide‐resistant genes.^[^ ^35^ ^]^ Virulence genes were also mostly enriched in the HOIT, including 5, 10‐ethylenetetrahydrofolate reductase polymorphisms, which act as potential risk factors for lung cancers, and the UDP‐N‐acetylglucosamine acyltransferase enzyme, which catalyzes the first reaction of lipopolysaccharide (LPS) biosynthesis (Figure 3f and Figure S5b, Supporting Information).^[^ ^36^, ^37^ ^]^

Similarly, we also observed differences in the phenotypes and functions of the salivary microbiome between the pneumo‐types. The proportion of saliva anaerobes in the HOIT group was significantly higher than that in the LOIT group (Wilcoxon test, *p* = 0.021). We profiled the genetic potential of the salivary microbiome using HUMAnN 2 (Figure S5c, Supporting Information) and found that 43 genes were differentially enriched, of which 33 were enriched in the HOIT and were mainly involved in the synthesis of viral proteins and transporters (Figure S5d, Supporting Information).

### 2.4 Significant Differences in Microbial Interaction Modes in the Oral Cavity and Lungs between Pneumo‐types

**Figure 4:**
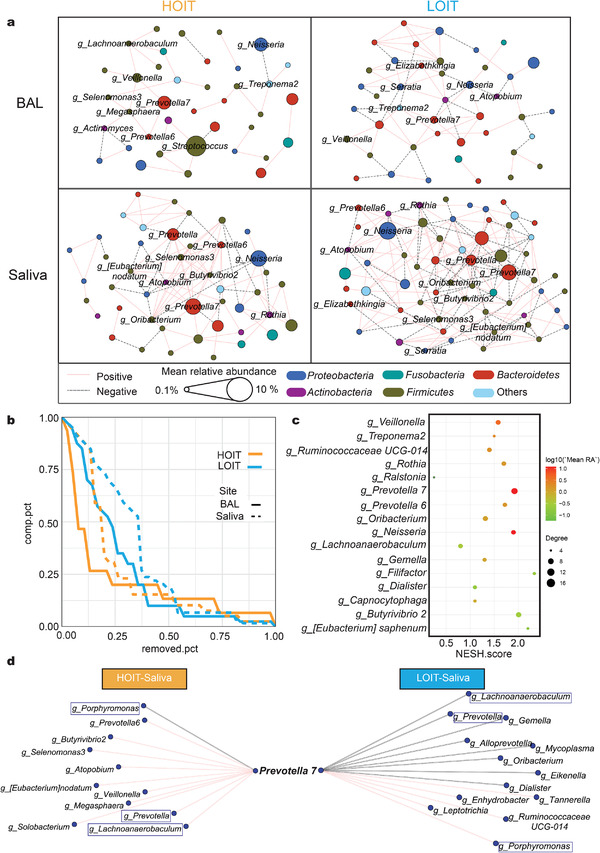
The interaction network of the BAL and saliva microbiome. a) A co‐occurrence network for genus‐level summarized taxa was built with SparCC as described in the Experimental Section. The four regions represent co‐occurrence networks of BAL (HOIT = 71, LOIT = 28) and saliva (HOIT = 59, LOIT = 22) samples in different pneumo‐types, respectively. The node colors represent the phyla to which the nodes belong. The node sizes represent the average relative abundance of the species. The colors of the lines represent interaction patterns. b) Robustness curves for the four networks. Attack robustness of a network was measured by sequentially removing nodes based on the nodes’ degrees selected and measuring the percentage of nodes that remained in the central connected component. Measurement of robustness was performed for each of our four networks and the results were plotted here with the percentage of nodes removed on the *X*‐axis and the percentage of remaining nodes in the central connected component on the *Y*‐axis. Each network is represented by a line on this graph. A larger area under the curve indicates a more robust network. The colors of the curves represent groups; the solid line BAL and the dashed line saliva. c) The nodes with significant differences in the network were screened by Netshift. The *X*‐axis represents NESH scores. The node sizes represent the degree numbers. The node colors represent the mean relative abundance. d) Co‐occurrence analysis highlighting the *Prevotella 7*‐interaction network in saliva. The taxa circled by the rectangles were common between the pneumo‐types. NESH: neighbor shift scores. Networks were produced by retaining edges (correlation coefficient *R* ranges between −0.4 and 0.4 and *p* < 0.05).

We selected the most abundant genera and used sparse correlations for compositional data algorithm (SparCC)^[^ ^38^ ^]^ to construct an ecological interaction network for the BAL and saliva microbiota of patients in the HOIT and LOIT groups, respectively (**Figure** **4**a). The degree numbers and average path lengths of the interaction networks of the LOIT were greater than those of the HOIT (Figure S6a, Supporting Information). To quantify the influence of taxon loss on network connectivity, we used Ruiz et al. to measure the “attack robustness” of the networks by sequentially removing nodes and measuring the size of the remaining largest connected components relative to its starting size.^[^ ^39^ ^]^ We observed that the area under the curve (AUC) in the LOIT was greater than that in the HOIT (Figure 4b), indicating that the network robustness of BAL and saliva in the HOIT was lower than that in the LOIT.

To assess the heterogeneous impact of oral microbes on the lung microbiota, we used Netshift^[^ ^40^ ^]^ to compare the saliva microbiome interaction network differences between pneumo‐types. *Prevotella 7*, *Prevotella 6*, *Neisseria*, *Butyrivibrio 2*, and another 12 genera were identified as critical nodes with high neighbor shift (NESH) scores, which meant that these genera contributed to the major differences between the two interacting networks, and were hence considered as “drivers” of the variations between the two pneumo‐types (Figure 4c and Table S3, Supporting Information). We selected the *Prevotella 7* subnetwork, which had the highest connection degree, to explore variations in interactions between these “drivers” and other microbes (Figure 4d). We observed that the *Prevotella 7* sub‐network in each pneumo‐type was significantly different (Fisher test *p* < 0.001), in which *Prevotella 7* was connected to 10 nodes (nine positive and one negative), whereas in the LOIT group, it was connected to 13 nodes (five positive and eight negative). We noted that three pairs of nodes that were positively correlated in the HOIT (*Prevotella 7* and *Porphyromonas*; *Prevotella 7* and *Prevotella*; *Prevotella 7* and *Lachnoanaerobaculum*) were all negatively correlated with the LOIT (Figure 4d).

**Figure 5:**
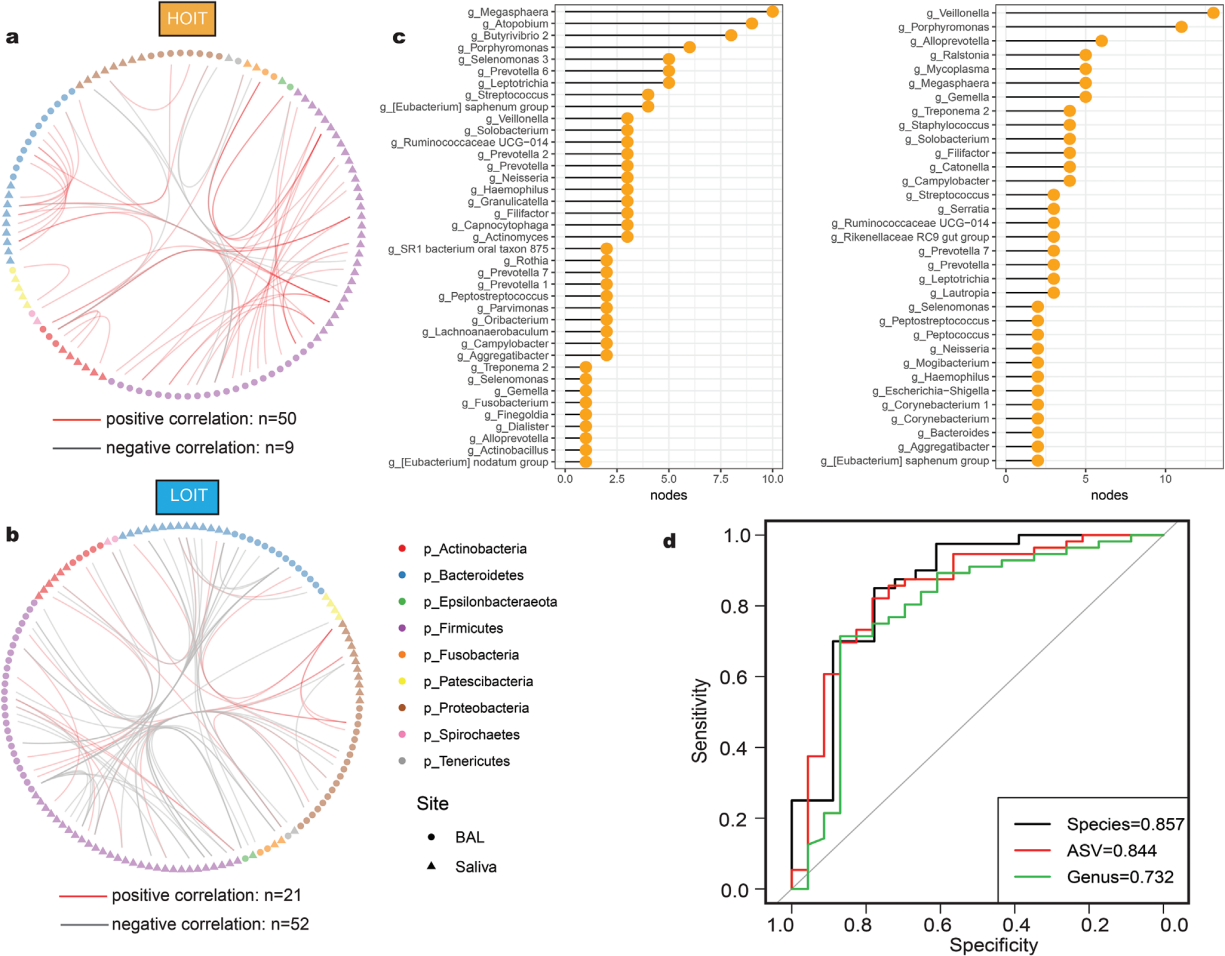
The characteristics of cross‐site interaction network between oral and lung microbiota. a,b) Network between oral and lung microbiota. The shapes of the nodes represent the body sites: triangle (saliva = 81) circle (BAL = 99). The colors of the nodes represent the phyla to which the nodes belong. The colors of the edges represent positive correlation (red) and negative correlation (gray). c) Key nodes in oral and lung communication network, and the values of the abscissa represent the sums of the numbers of edges of the nodes in the network. d) ROC curves of different classification levels. ROC: receiver operating characteristic.

Next, to further explore microbial interactions across body sites, we constructed an interaction network between the oral space and lungs for pneumo‐types and identified unique microbial interaction patterns specific to both. The ratio of positive correlations in the HOIT was greater than that in the LOIT (**Figure** **5**a,b). Then, we searched for important interaction nodes for both. In the HOIT, *Megasphaera*, *Atopobium*, and *Butyrivibrio 2* were important nodes. In the LOIT, *Veillonella* was vitally important (Figure 5c). These interaction network differences between the oral and lung microbiota of pneumo‐types suggest that the lung microbiota characteristics could be reflected through the oral cavity. Therefore, we evaluated the potential of saliva microbiota composition to predict pneumo‐types using a random forest approach. We constructed optimal models with input data from various taxonomic levels and found that the AUC reached 0.732, 0.844, and 0.857 for the genus, ASV, and species data, respectively (Figure 5d). Notably, in the ASV‐level random forest model, multiple ASVs from the *Prevotella 7* genus were included in the model variables (Table S4, Supporting Information).

### 2.5 Patients with either Pneumo‐Type Are Associated with Distinct Clinical Profiles

**Figure 6:**
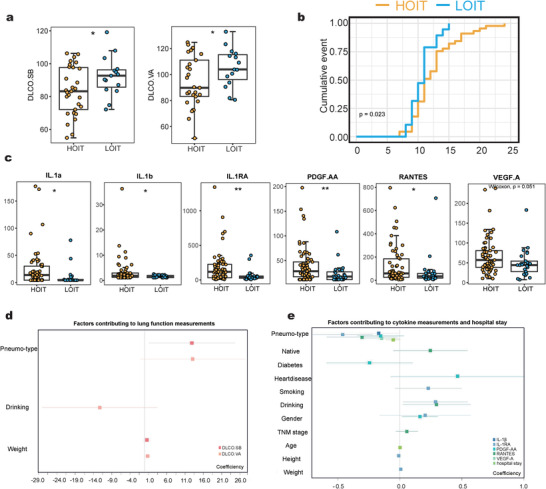
The cytokine levels of BALF and clinical data of patients. a) DLCO.SB and DLCO.VA were significantly different between the pneumo‐types (HOIT = 36, LOIT = 16). b) The *X*‐axis represents the length of hospitalization, and the *Y*‐axis represents the proportion of discharged patients at this time point. c) The boxplot of cytokines with significant differences between the pneumo‐types (HOIT = 61, LOIT = 25). d) The squares indicate the coefficients corresponding, and the horizontal lines indicate the 95% confidence intervals. Box plot: centerline, median; box limits, upper and lower quartiles; error bars, 95% CI. Differences between pneumo‐types were assessed using Wilcoxon test, * *p* < 0.05, ** *p* < 0.01, *** *p* < 0.001, **** *p* < 0.0001.

To determine whether penumo types corresponded to host health conditions, we investigated the clinical profiles of the cohort (Table S5, Supporting Information). We observed that the HOIT exhibited significantly lower DLCO.SB and DLCO.VA values (**Figure** **6**a, Wilcoxon test, *p* < 0.05) and longer hospital stays (Figure 6b) than the LOIT. The DLCO test is a noninvasive lung function test that measures the available surface area for gas exchange. The index usually decreases in different respiratory diseases, including emphysema, alveolar inflammation, and pulmonary fibrosis.^[^ ^41^ ^]^ We also observed variations in BAL cytokine levels between pneumo‐types (Figure 6c and Figure S7a, Supporting Information). Levels of interleukin (IL)‐1*α*, IL‐1*β*, IL‐1RA, PDGF.AA, and RANTES were significantly higher in the HOIT group (Figure 6c).

We constructed a linear regression model for each lung function measurement and cytokine level, using pneumo‐type and all demographic indices as variables. For each model, we calculated the model predictor and variance inflation factor (VIF). The results showed that the models had predictive performance (F‐test, *p* < 0.05), and the VIFs between the predictors were all lower than 4, indicating that all the calculated predictors were independent and not confounded by others (Figure 6d,e, also see Supporting Information, Table S6). As expected, the predictors calculated in each model included the pneumo‐type, indicating that the pneumo‐type is an important and irreplaceable determinant for the differences in lung function and cytokines among our cohort.

### 2.6 Oral Microbial Input Is Correlated with Lung Function and Cytokine Expression

**Figure 7:**
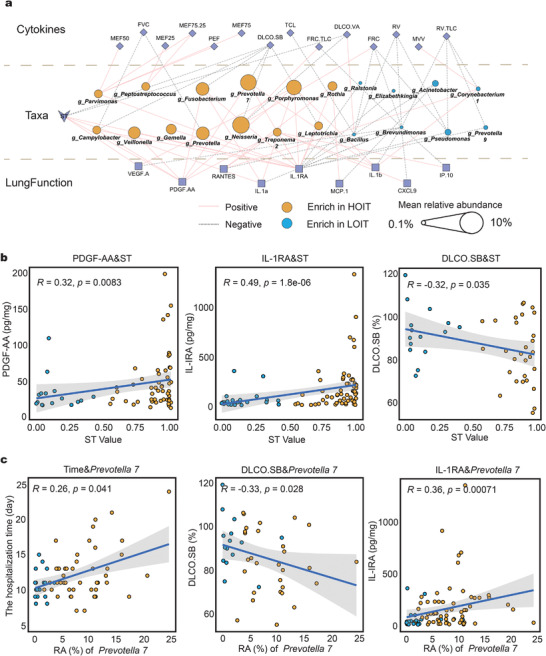
The correlation between the microbiota and cytokines or clinical data. a) We used LEfSe and ANCOM to pick out the genus with the most significant differences (LDA > 3.5) and calculated their Spearman correlation with cytokines and lung function. The colors of the lines indicate positive and negative correlations; node shapes represent variable attributes; the colors of the circles represent the pneumo‐types in which the genus was enriched; the sizes of the circles represent the mean relative abundance of the genera. b) Correlation scatter plots of ST values with partial lung function and cytokines. The lines represent the linear fitting curves and the shadows represent the confidence intervals of the fitting curves. c) Correlation scatter plots of *Prevotella 7* with hospitalization days, partial lung function and cytokines. The *p* and *R* values were calculated by Spearman correlation analysis. ST: SourceTracker value.

We constructed a correlation network for the differentially enriched microbes, cytokine levels, and lung function measurements. IL‐1RA was positively correlated with microbes enriched in the HOIT, but negatively correlated with those enriched in the LOIT (**Figure** **7**a). We also observed positive correlations*α* and IL‐1*β* and microbes enriched in the HOIT, including *Prevotella 7*, *Porphyromonas*, *Fusobacterium*, and *Treponema 2* (Figure 7a,b). Interestingly, the relative abundance of *Prevotella 7* was significantly and positively correlated with length of hospital stay (Figure 7c, Spearman *R* = 0.26, *p* = 0.041). In terms of lung function, *Neisseria*, one of the most abundant microbes in the HOIT, was negatively correlated with DLCO.SB and DLCO.VA (Figure 7a) and positively correlated with FRC and FRC/TLC.

We found that the SourceTracker value (ST) of saliva to BAL, which represents input intensity from oral to lung microbiota, was significantly and positively correlated with IL‐1*α*, IL‐1RA, VEGF‐A, and PDGF‐AA levels, all of which were elevated in the HOIT (Figure 7a,b, Spearman's *p<*0.05). Notably, the ST value was significantly but negatively correlated with DLCO.SB (Spearman's *R =* −0.32, *p* = 0.035) and DLCO.VA (Spearman's *R =* −0.35, *p* = 0.021) (Figure 7a).

## 3 Discussion

Here, we studied the processes by which oral and upper respiratory tract microbes shape the lung microbiota, focusing on how the heterogeneity of this shaping process is associated with interpersonal variation in lung microbiota and health. In particular, DLCO in the HOIT group was worse than that in the LOIT group, with proinflammatory cytokine (IL‐1*α*, IL‐1*β*) levels in the BAL also significantly increased. Interestingly, we observed concordance between oral and lung microbiota, characterized by overall synergism in the bacterial interaction network and niche differences represented by *Prevotella 7*. We also identified salivary microbiota features that may be used to predict lung microbiota status. These findings enabled us to better understand how saliva microbes impact the lung microbiota and increased the potential of developing saliva‐microbe‐based approaches to evaluate and regulate respiratory health.

The importance of the oral microbiome in human health is gaining traction.^[^ ^42^, ^43^ ^]^ The proximity and continuity of the oral cavity and lower respiratory tract allows the oral microbiome to be a potential determinant of the lung microbiome. Previous studies have reported similarities between lung and oral microbiota composition^[^ ^6^, ^9^ ^]^ showing that oral microbes enter the lower respiratory tract mainly through subclinical microaspiration.^[^ ^20^ ^]^ The oral microbiome may be the driving force underlying bacterial transformation by regulating mucosal immunity either directly or indirectly, thereby affecting pathogenicity.^[^ ^44^ ^]^ For instance, patients experiencing difficulties in swallowing and low awareness (for example, craniocerebral trauma patients) are more likely to develop pneumonia, which is usually caused by groups of bacteria in the oral cavity that accumulate in the lungs.^[^ ^45^ ^]^ In respiratory virus infections, the oral microbiota is closely related to severe acute respiratory syndrome coronavirus 2 (SARS‐CoV‐2) pulmonary infection, especially in patients with severe COVID‐19 whose lung hypoxia is more severe and conducive to the growth of anaerobes and facultative anaerobes from the oral microbiota. Therefore, oral health care is vital.^[^ ^46^ ^]^ While these findings revealed the comprehensive influence of the oral microbiome on the lungs, most studies were qualitative in nature and lacked evidence in clinical settings or applications. Therefore, we addressed this knowledge gap by measuring the intensity of oral microbial input into the lungs. We found that the intensity of communication between the oral and lung microbiota was closely related to lung cytokine levels and lung function.

We identified two lung microbiota community types (or pneumo‐types) according to their composition and oral input intensity. Healthy lung microbiota maintains a dynamic equilibrium between microbial immigration and elimination.^[^ ^4^, ^47^ ^]^ Changes in innate and adaptive immunity and physical lung anatomy may cause specific oral bacterial group migration into the lungs.^[^ ^48^ ^]^ Using the SourceTracker and DMM models, we identified HOIT and LOIT pneumo‐types and noted that the HOIT lung microbiota exhibited higher diversity with a higher abundance of resistance and virulence genes. Furthermore, we observed significant differences in specific cytokine expression levels between pneumo types. A previous study reported that PDGF‐AA regulates VEGF‐A expression during the transition from a precancerous lesion to advanced lung cancer.^[^ ^49^ ^]^ In our cohort, both PDGF‐AA and VEGF‐A levels were enriched in the HOIT group. RANTES and MCP‐1 constitute the C‐C class of the *β*‐chemokine supergene family. Both had inflammatory properties^[^ ^50^ ^]^ and were enriched in the HOIT.

We also observed differences in the composition and function of the salivary microbiota between pneumo‐types. The proportion of anaerobes in the HOIT saliva microbiota and the detection rate of synthetic genes related to bacterial virulence and adhesion were higher than those in the LOIT. Higher levels of human gamma herpes virus‐related genes were observed in the oral cavity of the HOIT. Several studies have reported that human gamma‐herpes virus accumulation in the lungs is closely related to the occurrence and development of specific lung diseases, such as idiopathic pulmonary fibrosis, asthma, and bronchiectasis.^[^ ^51^, ^52^ ^]^ Multiple genes related to ATP‐binding cassette (ABC) transporter and glycosaminoglycan binding protein synthesis were enriched in the oral microbiota of the HOIT and were involved in bacterial adhesion and biofilm synthesis,^[^ ^53^, ^54^ ^]^ suggesting saliva microbes in HOIT patients were more likely to colonize the lungs upon entry.

Our study provides a rationale for future diagnosis and intervention of lung microbiota by regulating or manipulating oral microbes. The oral cavity is a reservoir of respiratory and intestinal microbiota resources,^[^ ^55^ ^]^ and further studies may be necessary to explore the correspondence between the two microbial pneumo‐types and the highly individualized saliva microbiota seen in our study, as oral microbiota‐based lung diagnostics and lung interventions could be of considerable clinical importance. While previous investigators conducted comprehensive studies of the lung microbiota,^[^ ^8^, ^9^, ^20^, ^21^, ^22^, ^23^ ^]^ they largely overlooked the potential value of the oral microbiota. We identified evidence of co‐variation in oral and lung microbiota. The robustness of oral and lung bacterial interaction networks in the HOIT was lower than that in the LOIT, suggesting that the oral and lung microbiota in HOIT patients were less tolerant to external disturbances (sparse interactive networks were more susceptible to external interference).^[^ ^56^, ^57^ ^]^

The specific mechanisms underlying the formation of these pneumo‐types require further exploration, but our findings provide valuable insights. Our data suggest that oral microbes in the HOIT enter the lower respiratory tract more often and may be related to the disruption of the upper and lower respiratory barriers.^[^ ^58^ ^]^ Such situations are more common in elderly populations whose mucosal immune barriers are relatively vulnerable to aging, and oral bacterial enrichment is observed in this population's lungs.^[^ ^59^ ^]^ Changes in the host autogenic immune environment may also be responsible for pneumo‐types, as accumulated inflammatory factors in the respiratory tract are reportedly associated with microenvironmental changes in the respiratory tract.^[^ ^60^ ^]^

Our study has some limitations. We focused exclusively on the connections between the oral and lung microbiota. However, the human microbiome is a highly integrated ecosystem, and the interconnections between microbiota in different parts of the body, including different respiratory tract sites, should be studied in depth. We also observed that some rare genera, such as *Brevundimonas* and *Bacillus* in the nasal cavity, were not present in the oral cavity, but were involved in lung microbiota formation, suggesting an important role for these bacteria in shaping the lung microenvironment. This warrants further research. In addition, we were unable to repeat the BAL procedures on the same subjects and, as a result, no longitudinal analyses were conducted to monitor the dynamic characteristics of the oral and lung microbiota. Given the rhythms and periodicity of the oral microbiota and the lasting effects of oral input on lung microbiota, time‐series observations in experimental animals or human cohorts (using improved measurements) could comprehensively identify the co‐dynamics between oral and lung microbiota.

In summary, our data uncovered the microbial communication between the oral cavity and the lower respiratory tract and partially identified the association between pneumo‐types and respiratory health. Our work revealed the potential of using oral microbes as noninvasive sample alternatives to BAL to assess lung health, and also provides theoretical support for oral microbiota interventions to modulate lung microbiota.

## 4 Experimental Section

### Study Recruitment and Sample Collection

This study enrolled 67 patients with lung cancer and 32 healthy volunteers without lung disease. All samples were collected between January 23, 2019, and January 17, 2020. The enrolled participants, including lung cancer patients and volunteers without lung disease (details of study recruitment and sample collection are provided in the Supporting Information), all underwent BAL fluid, nasal swab, oropharyngeal swab, and saliva collection. To avoid possible contamination, samples were collected along with two empty tubes in parallel with specimen collection as negative controls.

### Clinical Information Records

Before bronchoscopy, clinical information was collected from each participant. Subsequently, demographic records were reviewed by two senior respiratory clinicians, with information gathered on height, weight, age, medical history, smoking history, drinking history, exposure history, genetic history, and antibiotic therapy within 6 months. Two researchers independently recorded and sorted all specific physiological and biochemical indicators of patients with lung cancer. These physiological and biochemical tests included complete blood counts, cardiac enzyme tests, emergency biochemical tests, lung function tests, liver function tests, and blood gas analyses.

### Lung Function Measurements

Lung function measurements directly indicated lung status. The MasterScreen/SentrySuite IOS system (CareFusion Co., California, US) was used to examine lung function. All lung function measurements, including forced vital capacity (FVC), forced expiratory volume in one second (FEV1), peak expiratory flow (PEF), maximal expiratory flow at 75% of the FVC (MEF75), maximal expiratory flow at 50% of the FVC (MEF50), maximal expiratory flow at 25% of the FVC (MEF25), maximal voluntary ventilation (MVV), total lung capacity (TCL), residual volume (RV), functional residual capacity (FRC), single‐breath diffusing capacity of the lung for CO (DLCO.SB), and DLCO.VA (DLCO divided by alveolar volume) were performed in accordance with protocols from the European Respiratory Society (ERS) and American Thoracic Society (ATS).

### Nucleic Acid Extraction and Metagenomic Sequencing

A DNA extraction control (extraction with only nucleic acid extraction reagent) and PCR control (extraction with only PCR reagents) were collected to further understand DNA extraction and possible contamination introduced during sequencing. In the following description and analysis, the sampling control, DNA extraction control, and PCR control are collectively referred to as negative controls.

All clinical samples, negative controls, and mock communities were stored at −80 °C until DNA extraction using a DNA Isolation Kit (Magigene, Guangdong). 16S rRNA sequencing was performed on V4 region amplicons with optimized primers: 515F, 5’‐GTGCCAGCMGCCGCGGTAA‐3’; 806R, and 5’‐GGACTACHVGGGTWTCTAAT‐3’ on an Illumina HiSeq2500 platform. Primers were synthesized by Invitrogen (Carlsbad, CA). PCR reactions, containing 25 µL 2x Premix Taq (Takara Biotechnology, Dalian Co. Ltd., China), 1 µL of each primer (10 × 10^−3^ m), and 3 µL DNA (20 ng µL^−1^) template in a volume of 50 µL, were amplified by thermocycling: 5 min at 94 °C for initialization; 30 cycles of 30 s denaturation at 94 °C, 30 s annealing at 52 °C, and 30 s extension at 72 °C, followed by 10 min final elongation at 72 °C. Three replicates were done per sample and each PCR product from the same sample were mixed with the BioRad S1000 (Bio‐Rad Laboratories, CA).

Negative control samples showed low concentrations after 16S rRNA gene PCR amplication (<10 ng µg^−1^, Figure S1a, Supporting Information), and did not meet the concentration criteria for regular sequencing library preparation. However, to further understand the contamination that might be introduced during sample collection and sequencing, sequencing library preparation was conducted for these negative control samples. The 16s rRNA gene of 99 BAL samples, 87 oropharyngeal swabs, 86 nasal swabs, and 81 saliva samples was sequenced successfully, producing a total of 45 150 202 reads (mean ± SD per sample: 127 904 ± 60 718). At the same time, the four negative controls had fewer sequence reads than the clinical samples (mean ± SD per sample: 17652 ± 12703) (Table S7, Supporting Information).

High‐quality DNA samples from BAL (*n* = 55) and saliva (*n* = 58) samples were used for metagenomic sequencing. Standard bacterial genomic DNA mixes (mock communities) were also submitted for sequencing as positive controls. Sequencing libraries were generated using the NEB Next Ultra DNA Library Prep Kit (NEB, USA) on the Illumina platform, and index codes were added to assign sequences to the samples. Sample DNA was fragmented by sonication to ≈300 base pairs (bp), and fragments were end‐polished and ligated with full‐length adaptors for Illumina sequencing with further PCR amplification. The libraries were analyzed for size distribution using an Agilent 2100 Bioanalyzer (Agilent, USA) and then sequenced on the Illumina NovaSeq 6000 platform.

### 16S rRNA Gene Sequence Analysis

The 16S rRNA gene sequences were analyzed using the Quantitative Insights into Microbial Ecology (QIIME2 2019.6) pipeline for microbiome data.^[^ ^61^ ^]^ Raw sequence data were de‐multiplexed and quality‐filtered using the q2‐demux plugin, followed by denoising with DADA2.^[^ ^62^ ^]^ All ASVs were aligned with mafft^[^ ^63^ ^]^ and used to construct a phylogenetic tree using fasttree2.^[^ ^64^ ^]^ Taxonomy was assigned to ASVs using the q2‐feature‐classifier classify‐sklearn naive Bayes taxonomy classifier against SILVA‐132 at a cut of 99% sequence similarities. BugBase^[^ ^34^ ^]^ was used to predict bacterial composition based on the sequencing results.

### Evaluation of Negative Controls and Mock Community Sequencing Results

Two sets of mock communities were used in this study. Mock community 1 contained 48 species of bacteria from 20 genera, and mock community 2 contained seven species of bacteria from seven genera. Only 0.80% and 0.83% of sequences were found outside the set of sequences in the sequencing results, indicating that the final sequencing results of the mock communities were highly consistent with the mock standards (Figure S1b, Supporting Information). The top five ASVs in the sampling negative controls and the top five ASVs in the DNA extraction and PCR controls accounted for a very low proportion (<1%) in the BAL samples (Table S8, Supporting Information). These data suggest that the sequencing process did not cause serious pollution in the samples, so no taxa were eliminated in subsequent analyses.

### Shotgun Metagenomic Analysis

The first crucial step in metagenomic analysis is quality control and removal of host contamination from raw reads. The KneadData (v0.7.5) pipeline or a combination of Trimmomatic^[^ ^65^ ^]^ (v0.33) and Bowtie2^[^ ^66^ ^]^ (v2.2.3) was used. Trimmomatic was run with the following default arguments: “SLIDINGWINDOW:4:20 MINLEN:70.” The minimum length was computed as 70% of the input read length. GRCH38 was used as the host reference genome. Kraken2^[^ ^67^ ^]^ (v2.0.7) was used to align the reads from each sample against the constructed database. Reads aligned to multiple database entries were assigned to their last common ancestor in the taxonomic tree. Reads assigned to internal taxonomical nodes were reassigned by Braken^[^ ^68^ ^]^ (v2.5.0) using the number of reads from all samples with unique mapping to the database. ShortBRED^[^ ^69^ ^]^ (v0.9.4) was used to quantify the abundance of antibiotic resistance and virulence genes. ShortBRED markers were identified from the annotated antibiotic resistance or virulence proteins using the reference database of PRE‐COMPUTED SHORTBRED MARKERS. Clean reads were mapped against marker sequences with 99% sequence identity. All analyses were performed on gene abundance normalized to reads per kilobase per million (RPKM). Functional profiling was performed with HUMAnN2^[^ ^70^ ^]^ v0.11.2, which involved mapping post‐processed reads against the pangenomes of detected species, allowing read‐count‐based quantification of microbial gene families in samples. The identified gene families were further mapped to the MetaCyc database to quantify the metabolic pathways. Both the gene families and pathway profiles were stratified according to the contributing organisms. For each sample, the gene richness was calculated by counting the number of unique gene families present.

### Neutral Model and NTS Analyses

To determine the relative importance of neutral processes and selection in the lung microbiome, the version of the neutral model was implemented using custom scripts in R (Supporting Information). The normalized stochasticity ratio was calculated using the R package NST.^[^ ^71^ ^]^

### Cytokine Measurements in BAL Fluid

For the cytokine assays, 87 participants were analyzed who provided a sufficient number of BALF samples. A total of 48 cytokines were measured in the concentrated BAL using the Luminex Human Cytokine/Chemokine/Growth Factor Panel (Millipore: HCYTA‐60K) according to the manufacturer's instructions in a MAGPIX system (Luminex). These included soluble CD40 ligand (sCD40 L), epidermal growth factor (EGF), fibroblast growth factor‐2 (FGF‐2), Eotaxin, Flt‐3 ligand, fractalkine, granulocyte colony‐stimulating factor (G‐CSF), granulocyte‐macrophage colony‐stimulating factor (GM‐CSF), growth‐related oncogene‐*α* (GRO), interferon alpha‐2/*γ* interleukin (IFN‐*α*2/IFN‐*γ*), (IL)‐1 receptor antagonist (IL‐1RA), IL‐1*α*, IL‐1*β*, IL‐2, IL‐3, IL‐4, IL‐5, IL‐6, IL‐7, IL‐8 (CXCL8), IL‐9, IL‐10, IL‐12 (p40), IL‐12 (p70), IL‐13, IL‐15, IL‐17A, IL‐17E, IL‐17F, IL‐18, IL‐22, IL‐27, interferon *γ*‐induced protein 10 (IP‐10), monocyte chemotactic protein 1/3 (MCP‐1/MCP‐3), macrophage colony‐stimulating factor (M‐CSF), macrophage‐derived chemokine (MDC), monokine induced by gamma interferon (MIG), macrophage inflammatory protein 1*α*/1*β* (MIP‐1*α*/MIP‐1*β*), platelet derived growth factor‐AA/‐AB (PDGF‐AA/PDGF‐AB), regulated upon activation (RANTES), transforming growth factor alpha (TGF‐*α*), tumor necrosis factor *α*/*β* (TNF‐*α*/TNF‐*β*), and vascular endothelial growth factor‐A (VEGF‐A). The average results from technical duplicates were used. Cytokines detected in less than one‐third of the samples were not included in the analyses: FLT.3L, GM. CSF, IFN‐a2, IFN‐*γ*, IL‐2, IL‐3, IL‐4, IL‐12 (p70), IL‐17E, IL‐17E, IL‐17F, IL‐22, MCP‐3, PDGF‐AB, and TNF‐*α*. Data were analyzed using MILLIPLEX Analyst.V5.1 software.

### Statistical Analysis

A nonparametric Spearman's correlation test was used to test the associations between continuous variables. The false discovery rate (FDR) was used as a control for multiple tests. Linear discriminant analysis effect size (LEfSe)^[^ ^72^ ^]^ was used to identify differential bacteria between pneumo‐types, and ANCOM^[^ ^73^ ^]^ was used to validate the results; only the overlapping results were subjected to downstream analysis. Visualization of evolutionary trees was performed using the R package ggtree.^[^ ^74^ ^]^ Differences in microbial communities were calculated using the Bray‐Curtis dissimilarity index and visualized using a PCoA ordination plot. Differences in community composition were statistically assessed using permutational multivariate analysis of variance (PERMANOVA)^[^ ^75^ ^]^ with Bonferroni correction for multiple comparisons. Alpha diversity was calculated as the Shannon index using the vegan package.^[^ ^76^ ^]^

Random forest models were constructed with the R package AUCRF (v.1.1)^[^ ^77^ ^]^ using the three types of compositional data (genus‐level, ASVs, and species) as input. Optimal predictors were determined based on the mean decrease in the accuracy of the model in classifying the subjects. SourceTracker^[^ ^32^ ^]^ (v0.9.8, default parameters) was used to assess the migration of the donor microbiota. The bacterial community types were determined using a DMM algorithm‐based method.^[^ ^33^ ^]^ Microbes with more than 1000 reads were selected as core taxa to construct the interaction network using SparCC,^[^ ^38^ ^]^ and 1000 bootstraps were used to calculate correlations and *p* values. Networks were produced by retaining edges (correlation coefficient *R* ranges between −0.4 and 0.4 and *p* < 0.05), analyzed in R with the package igraph^[^ ^78^ ^]^ and visualized with Cytoscape 3.8.1.^[^ ^79^ ^]^

A linear model was constructed to test the associations between phenotypic data (dependent variables), such as lung function and cytokines, and independent variables, such as pneumo‐type, native, college, diabetes, hypertension, heart disease, gastroenteropathy, smoking, drinking, gender, TNM stage, age, height, weight, and BMI. For data pre‐processing, Shapiro tests^[^ ^80^ ^]^ were performed on the dependent variables to determine whether they conformed to a normal distribution. Since the cytokine indices did not conform to a normal distribution, log‐transformations of the cytokine indices were performed. Then, Spearman correlation analysis on these independent variables and dependent variables was performed to screen out the independent variables that were significantly correlated with dependent variables and constructed a linear model with lung function and cytokines as dependent variables.

### Ethics Approval and Consent to Participate

All patients provided written informed consent in accordance with the Declaration of Helsinki and local ethics committee. This study was registered with the Chinese Clinical Trial Registration Center (registration no. ChiCTR1900023317, 2019/5/22) and was approved by the Research Ethics Committee of the Fifth Affiliated Hospital of Sun Yat‐sen University (Approval No. K51‐1, 2018).

## Conflict of Interest

The authors declare no conflict of interest.

## Author Contributions

J.X.Z., Y.P.W., J.L., and Y.Q.Y contributed equally to the study. G.‐B.T. and T.D. conceived, designed, and supervised the study. J.X.Z., Y.P.W., H.L., and Y.J.L. performed the experiments and produced tables and figures. Y.Q.Y. and X.R.W. contributed to the data analysis and interpretation. J.X.Z., X.R.W., X.B.Z., Y.J.L., C.L.T., M.Z.C., C.Y.T., B.Z.C., Y.Y.H., and Z.G.W. participated in the sample collection, clinical data, and experiments. J.X.Z., Y.P.W., Y.Q.Y., and H.L. searched for relevant literature. J.X.Z. and Y.P.W. wrote the manuscript, which was reviewed and edited by G.‐B.T. and T.D. All authors have reviewed, revised, and approved the final manuscript.

## Supporting information

Supporting Information

Supplemental Table 1

Supplemental Table 2

Supplemental Table 3

Supplemental Table 4

Supplemental Table 5

Supplemental Table 6

Supplemental Table 7

Supplemental Table 8

## Data Availability

The data that support the findings of this study are openly available in PRJNA757607 and PRJNA757846 at https://www.ncbi.nlm.nih.gov/bioproject/PRJNA757846 https://www.ncbi.nlm.nih.gov/bioproject/PRJNA757607.
