# Supplementary material for: Differential Oral Microbial Input Determines Two Microbiota Pneumo‐Types Associated with Health Status
Source: Adv Sci (Weinh). 2022 Aug 28;9(32):2203115. doi: 10.1002/advs.202203115 (PMC9661847; doi:10.1002/advs.202203115)
Supplement: Supplementary file 1 — Supporting Information [file ADVS-9-2203115-s002.pdf]

ADVANCED SCIENCE
Open Access

Supporting Information

for *Adv. Sci.*, DOI 10.1002/advs.202203115

Differential Oral Microbial Input Determines Two Microbiota Pneumo-Types Associated with Health Status

Jingxiang Zhang, Yiping Wu, Jing Liu, Yongqiang Yang, Hui Li, Xiaorong Wu, Xiaobin Zheng, Yingjian Liang, Changli Tu, Meizhu Chen, Cuiyan Tan, Bozhen Chang, Yiying Huang, Zhengguo Wang, Guo-Bao Tian and Tao Ding**

Supporting Information

Oral bacteria contribute to respiratory health by shaping lung microbiota

Jingxiang Zhang[1,2†], Yiping Wu[2,3†], Jing Liu[4†], Yongqiang Yang[2,3†], Hui Li[1,2], Xiaorong Wu[1,2], Xiaobin Zheng[4], Yingjian Liang[4], Changli Tu[4], Meizhu Chen[4], Cuiyan Tan[4], Bozhen Chang[1,2], Yiying Huang[4], Zhengguo Wang[4], Guobao Tian[2,3,5,a], Tao Ding[1,2,b*]*

Affiliations

[1]Department of Immunology, Zhongshan School of Medicine, Sun Yat-sen University, Guangzhou 510080, China

[2]Key Laboratory of Tropical Diseases Control (Sun Yat-sen University), Ministry of Education, Guangzhou 510080, China

[3]Department of Microbiology, Zhongshan School of Medicine, Sun Yat-sen University, Guangzhou 510080, China

[4]Department of Respiratory Medicine, The Fifth Affiliated Hospital of Sun Yat-sen University, Zhuhai 519000, China

[5]School of Medicine, Xizang Minzu University, Xianyang, Shaanxi 712082, China.

[a]ORCID: 0000-0001-6750-5482

[b]ORCID: 0000-0002-2200-0496

[†]These authors contributed equally to this article.

[*]These senior authors contributed equally to this article as corresponding authors.

Supplementary Methods

Neutral Model

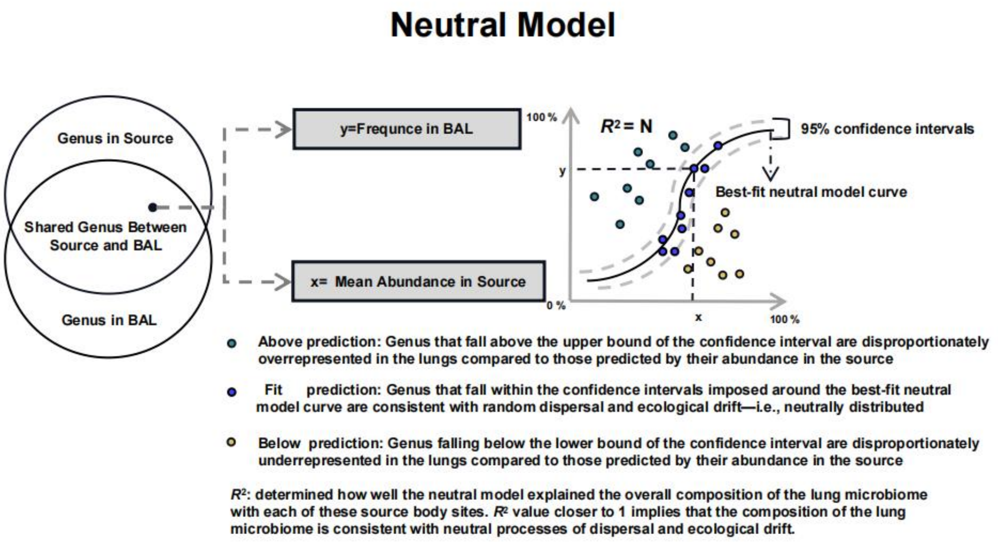

The fitting of this parameter was performed in R using non-linear least-squares fitting and the minpack.lm package. The goodness of fit of this curve was assessed using the coefficient of determination (R^2). Binomial proportion 95% confidence intervals around the model predictions were calculated using the Wilson score interval in the HMisc package in R.

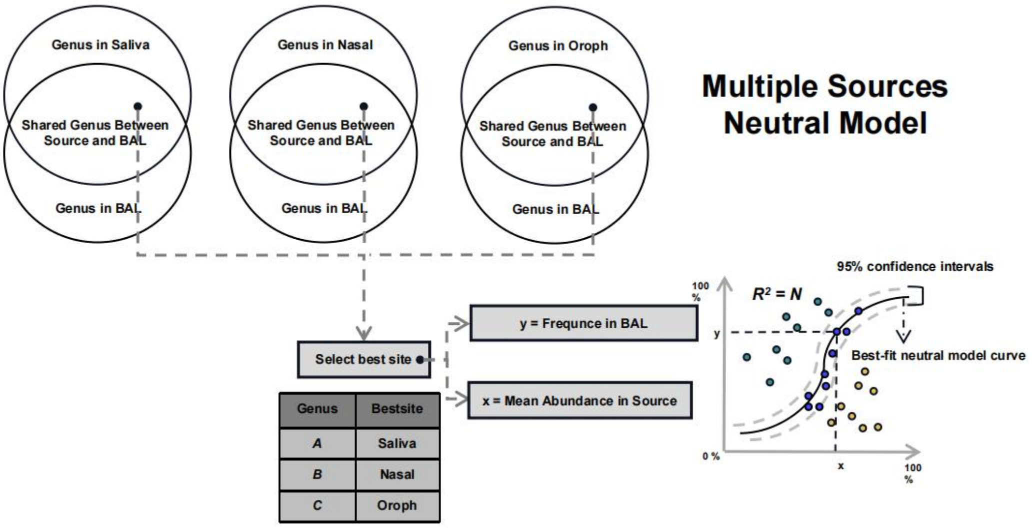

Study recruitment and sample collection

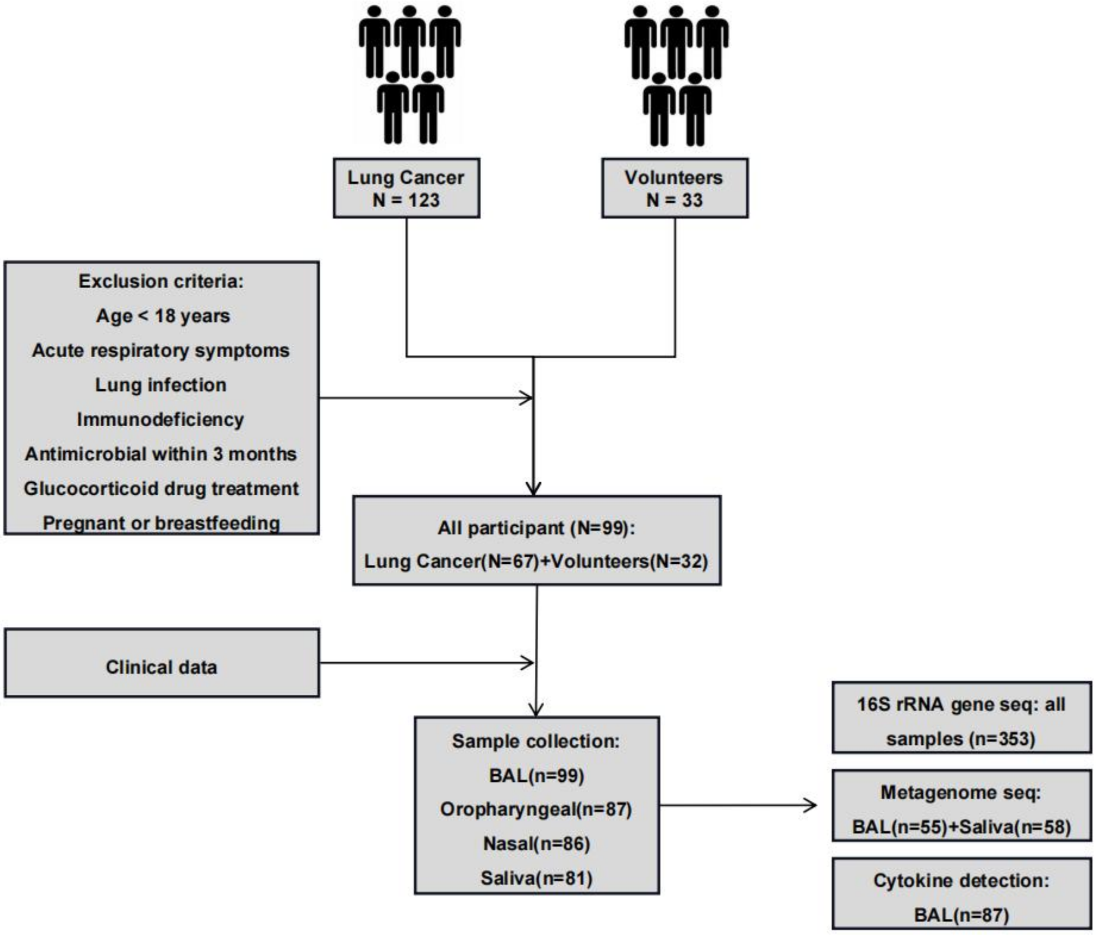

The cohort inclusion criteria for this study are as follows: age > 18 years, no acute respiratory symptoms; not receiving antimicrobial within 3 months before screening or glucocorticoid drug treatment; not immunodeficiency; not pregnant or being breastfeeding. Patients who had taken any antibiotics in the last three months were excluded since antibiotics exert significant effects on the microbiome in the respiratory tract and other sites. Except for lung cancer and three lung cancer patients with bronchiectasis, participants had no other respiratory complications.

The inclusion criteria for lung cancer patients are as follows: lung cancer was diagnosed by histopathology in the lung intensive care unit (PCCM), thoracic surgery and oncology department, without lung infection and other respiratory symptoms. The control participants were volunteered through online registration on the website of the Fifth Affiliated Hospital of Sun Yat-sen University, and were followed up by telephone call and on-site screening to assess the condition of respiratory symptoms

and family history of respiratory disease. All participants have fully understood the operation situation and signed the informed consent form. The clinical samples were taken two to three days after admission, during which time the patient received no antibiotics or corticosteroids but blood oxygen monitoring, temperature and blood pressure measurements, and routine in-patient care.

The enrolled 67 lung cancer patients and 32 volunteers without lung disease all underwent bronchoalveolar alveolar lavage fluid, nasal swab, oropharyngeal swab and saliva collection. When bronchoscopy is performed for alveolar lavage, respiratory specialists with an over 5-year experience in bronchoscopy used a nasal approach for research. We avoided suctioning until our scope reaches the position for sampling as possible, as the underlying carry-over of upper airway microorganism to the lung. Approximately 60 ml of sterile saline in total was passed through the scope channel and about 20-30 ml bronchoalveolar lavage (BAL) was recovered. Samples were immediately frozen at 4 degrees Celsius. Nasal swabs, oropharyngeal swabs, and saliva were collected on the day of bronchoscopy. When collecting nasal swabs and throat swabs, use a sterile cotton swab to rotate the corresponding body part of the subject at least five times. All swabs collected were immediately frozen at -80 degrees celsius. Saliva collection requires subjects to perform before bronchoscopy. Approximately 5 mL of saliva is collected and frozen immediately.

Supplemental Figures

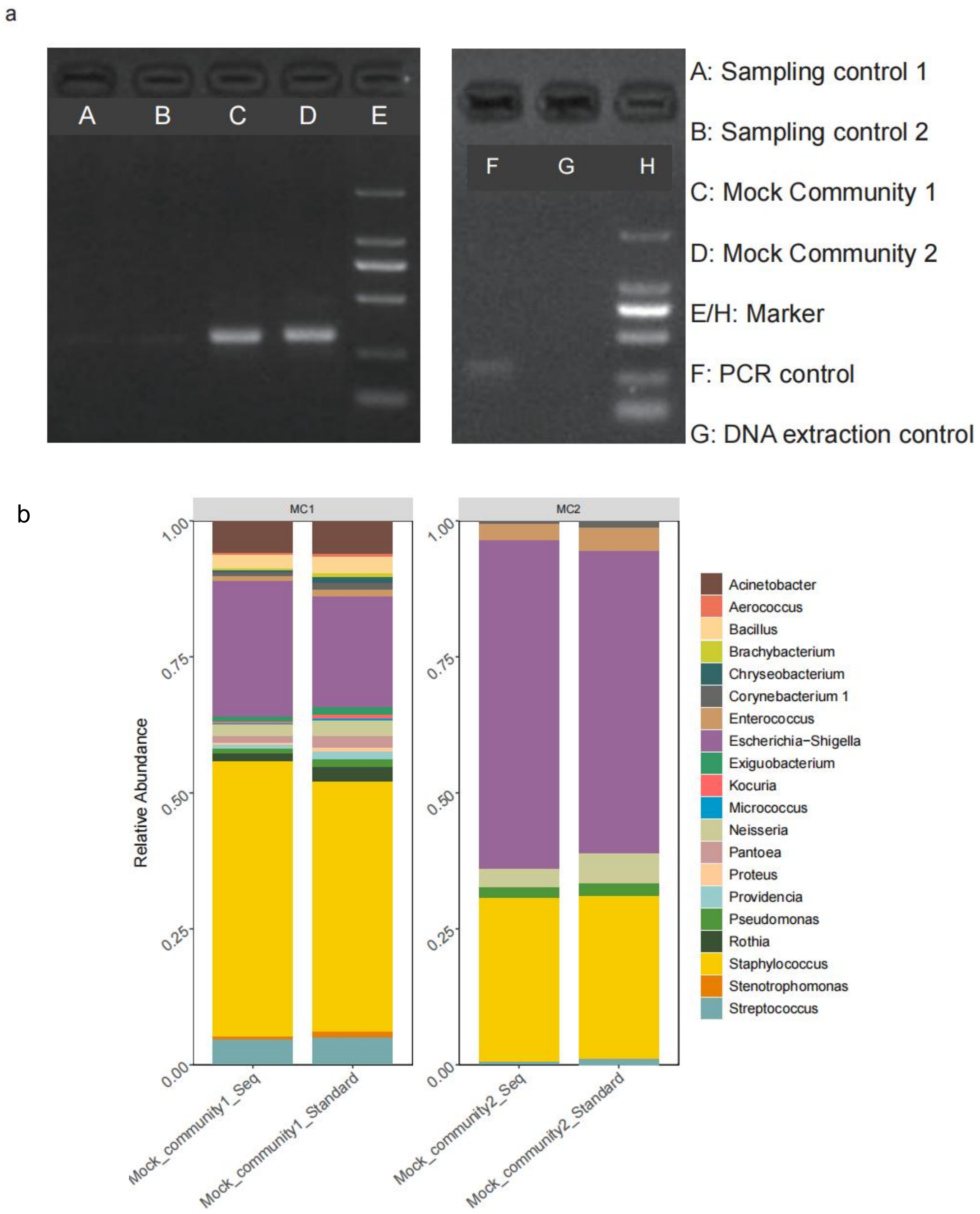

Figure S1. (a) The agarose gel electrophoresis result of PCR products of negative controls and mock communities. **(b)** Comparison of mock community sequencing results with standard results.

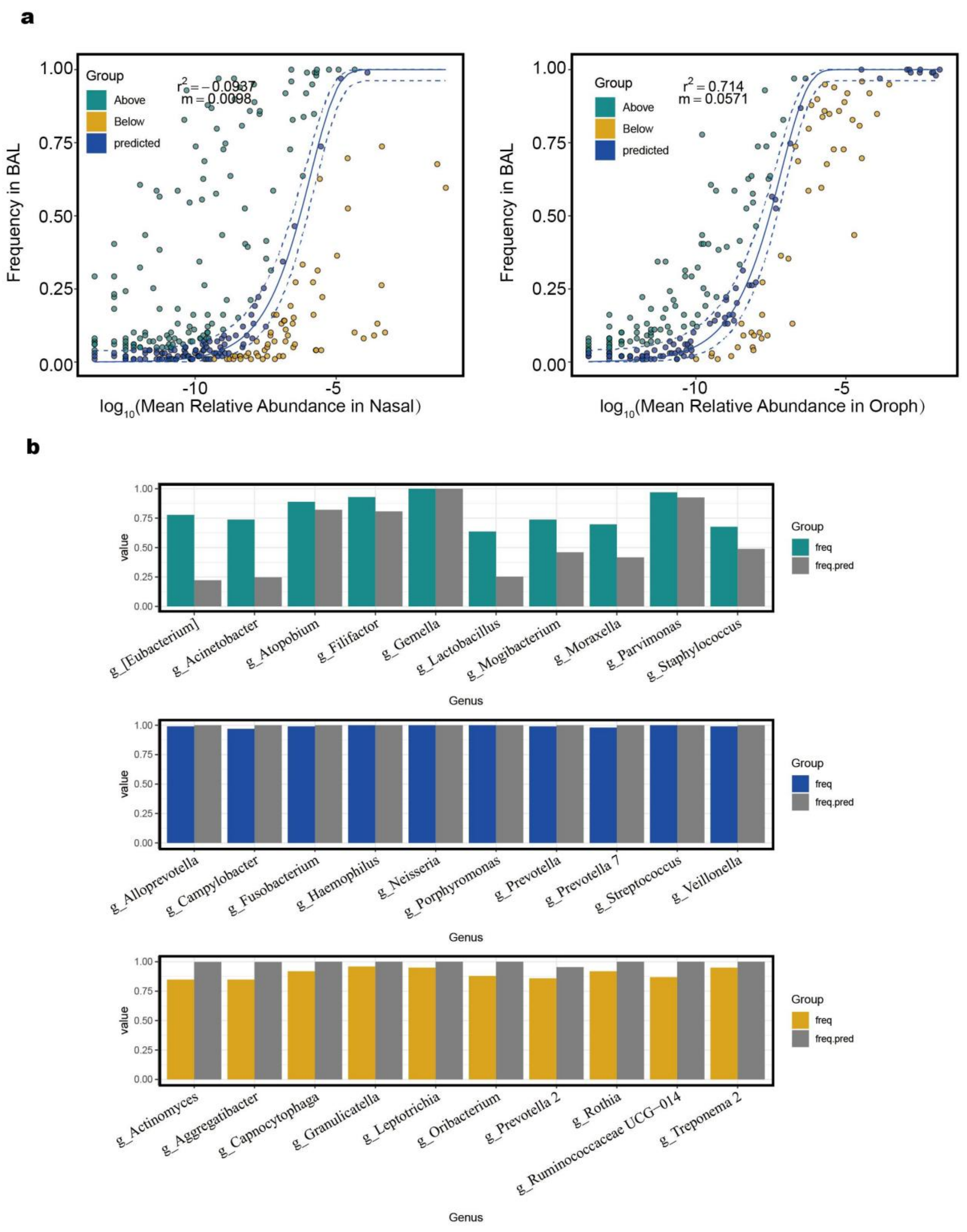

Figure S2. **(a)** Neutral model of nasal (left) an-d oropharynx (right) with BAL. **(b)** 9 Bacteria that conform to and deviate from the neutral model (Ten bacteria with the 90 highest detection frequency in BAL were selected).

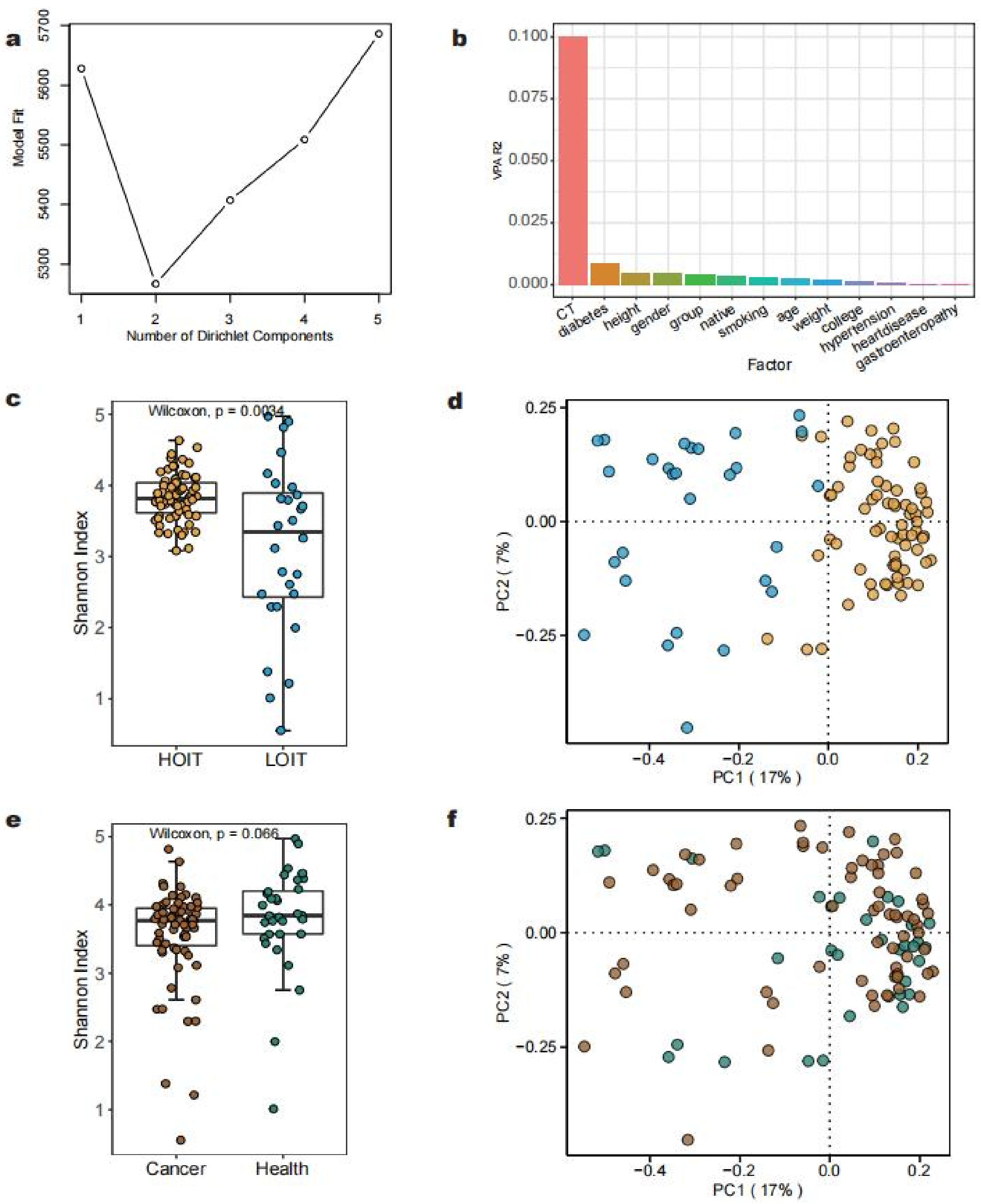

Figure S3. (a) Evaluates model fit for increasing number of Dirichlet mixture components using the laplace approximation to the negative log model evidence. (**b**) VPA results showed that pneumo-type was the most important explanatory factor of lung microbiome heterogeneity **(c,e)** The lung microbiome α diversity of lung cancer patients (n=67), volunteers (n=32), LOIT (n=28), HOIT (n=71). **(d,f)** PCoA based on Bray-Curtis demonstrate that there was no difference in microbial composition between lung cancer patients and volunteers. Differences between groups were assessed using Wilcoxon test, * $p < 0.05$, ** $p < 0.01$, *** $p < 0.001$, **** $p < 0.0001$. VPA: Variance Partitioning Analysis.

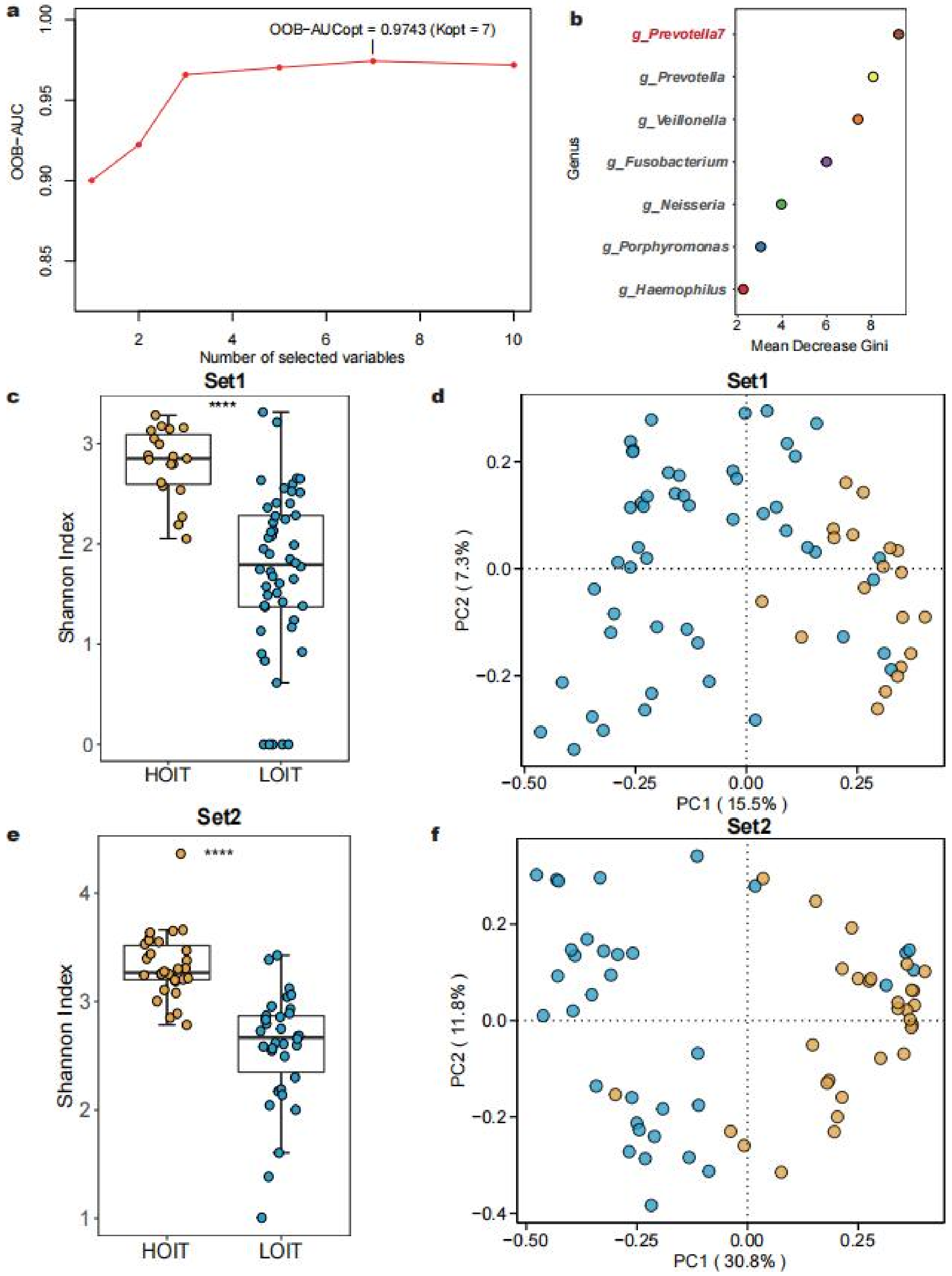

Figure S4. (a) The optimal number of variables required to construct a random forest model. (**b**) The importance ranking results of selected variables in the construction of a random forest model. **(c,e)** Public data sets were classified by random forest model and α diversity was compared (Set1: HOIT=19, LOIT=50;Set2: HOIT=30, LOIT=34). (**d,f**) Public data sets were classified by random forest model and β diversity was compared. Differences between groups were assessed using Wilcoxon test, * $p < 0.05$, ** $p < 0.01$, *** $p < 0.001$, **** $p < 0.0001$.

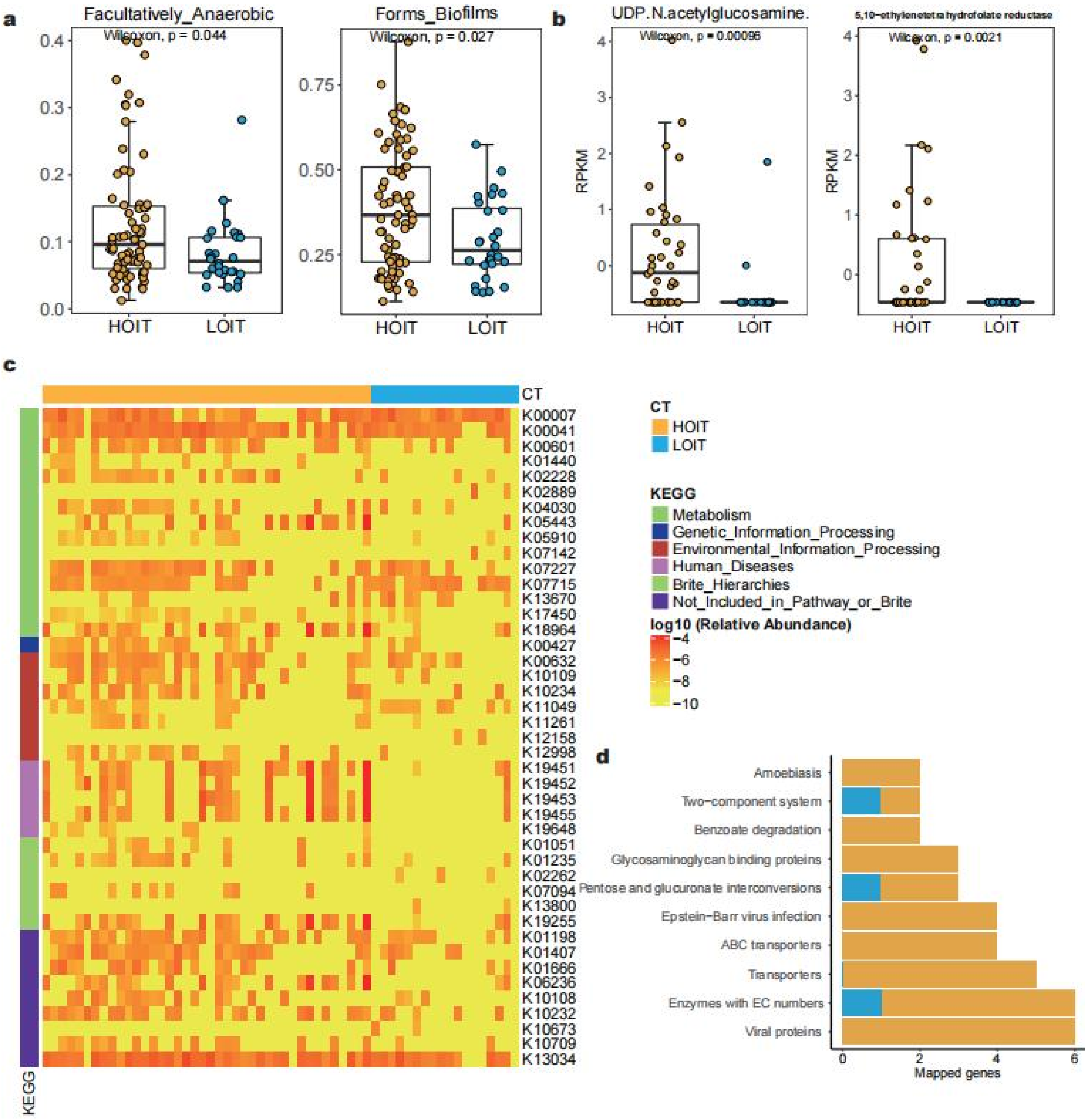

Figure S5. (**a**) BAL microbiota in the HOIT (n=40) group showed more facultative anaerobe and stronger biofilm-forming ability than LOIT (n=18). **(b)** UDP-N-acetylglucosamine acyltransferase enzyme gene and 5, 10-ethylenetetrahydrofolate reductase gene were enriched in HOIT group. **(c)** Heatmaps of functional genes with differences in the saliva microbiota of the two pneumotypes. **(d)** KEGG pathway mapping of the genes in a, indicating enriched functional pathways. Differences between groups were assessed using Wilcoxon test, * $p < 0.05$, ** $p < 0.01$, *** $p < 0.001$, **** $p < 0.0001$.

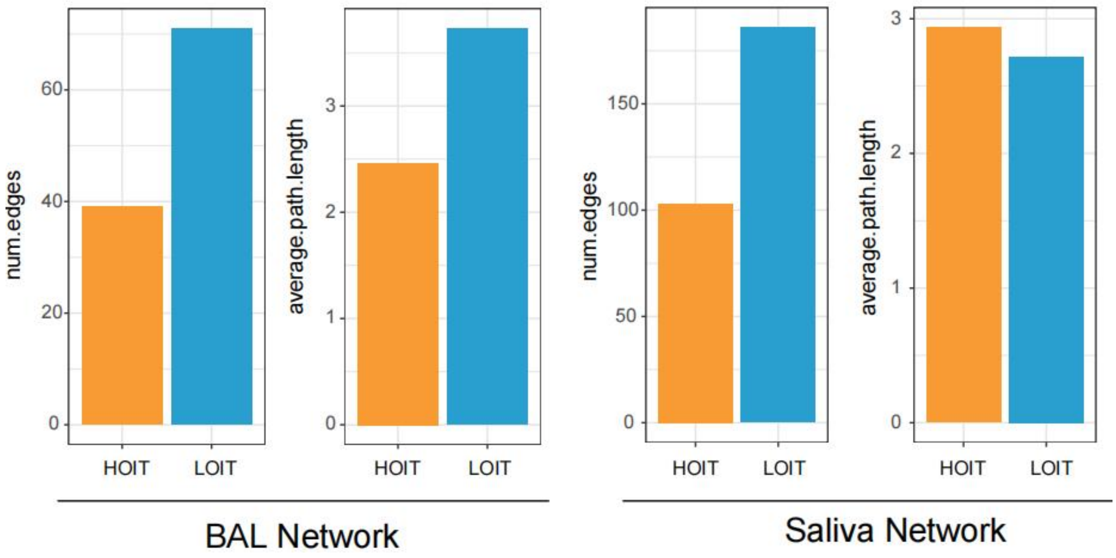

Figure S6. (a) The number of network nodes and the comparison of the average path length.

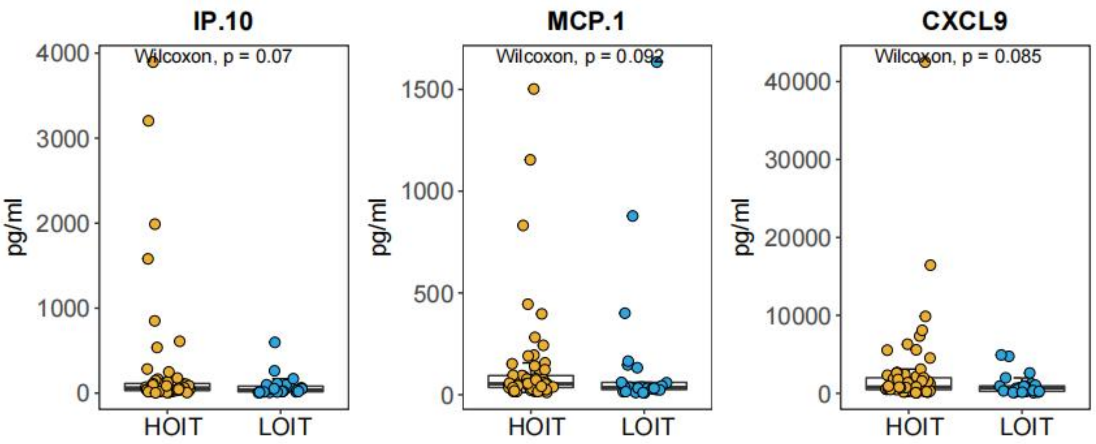

Figure S7. (a) The pro-inflammatory and pro-oncogenic cytokines were enriched in 1 HOIT (n=61). Differences between groups were assessed using Wilcoxon test, * p < 0.05, ** p < 0.01, *** p < 0.001, **** p < 0.0001.

Supplemental tables

Table S1

The results of pneumo-types in 99 patients. ID is the sample name, SourceTracker group is the grouping after high or low grouping based on saliva contribution value, and DMM group is the grouping based on DMM model.

Table S2

The results of LEfSe and ANCOM.

Table S3

The results of Netshift analysis of salivary microbiota interaction network. N (LOIT/HOIT) is the number of node degrees in LOIT/HOIT, Exclusive is unique in HOIT, DelBet is the delta value from LOIT to HOIT, and COM is the community membership degree of the node in HOIT.

Table S4

Random forest model selects specific taxas at species level, ASV level and genus level.

Table S5

The demographic and clinical data statistics.

Table S6

The results of linear model fitting.

Table S7

The basic statistics of sequencing data.

Table S8

The relative abundance of dominant ASVs in negative controls and clinical samples.
